# Alpha-fetoprotein level to total tumor volume as a predictor of hepatocellular carcinoma recurrence after resection. A retrospective cohort study

**DOI:** 10.1016/j.amsu.2020.04.014

**Published:** 2020-05-04

**Authors:** Hazem M. Zakaria, Anwar Mohamed, Hazem Omar, Nahla K. Gaballa

**Affiliations:** a Department of Hepatopancreatobiliary and Liver Transplant Surgery, National Liver Institute, Menoufia University, Egypt; b Department of Hepatology and Gastroenterology, National Liver Institute, Menoufia University, Egypt; c Department of Diagnostic and Intervention Radiology, National Liver Institute, Menoufia University, Egypt; d Department of Anesthesiology and Intensive Care, National Liver Institute, Menoufia University, Egypt

**Keywords:** Hepatocellular carcinoma, Alfa fetoprotein, Total tumor volume, Liver resection

## Abstract

**Background:**

Total tumor volume (TTV) and serum alfa fetoprotein (AFP) level are important risk factors linked with the high possibility of hepatocellular carcinoma (HCC) recurrence. The aim of the study was to evaluate the role of AFP/TTV ratio, as a prognostic marker, in the prediction of HCC recurrence after resection.

**Methods:**

Patients who underwent liver resection for HCC between 2010 and 2018 were retrospectively analyzed. Patients were divided into 2 groups; a group with AFP/TTV ≤2 and another group with AFP/TTV >2. Risk factors for HCC recurrence were recorded.

**Results:**

A total of 286 HCC patients underwent liver resection (184 patients with AFP/TTV ≤ 2, and 102 patients with AFP/TTV > 2). There was a significant difference between the 2 groups in the preoperative total bilirubin level, serum AFP level, mean tumor diameter, TTV, operative blood loss, microvascular invasion and hospital stay (all *P* values < 0.05). The 1-, 3-, and 5-year tumor recurrence rates were 24.1%, 43%, and 57.6% respectively. The independent risk factors for tumor recurrence were AFP/TTV >2 (HR = 1.62, 95% CI = 1.29–1.98, *P* = 0.042), Macrovascular invasion (HR = 2.03, 95% CI = 2.17–2.38, *P* = 0.021, and microvascular invasion (HR = 1.36, 95% CI = 1.08–1.77, *P* = 0.019).

**Conclusion:**

AFP/TTV ratio is a feasible prognostic marker for prediction of HCC recurrence after resection so, it can help in providing an intensive postoperative surveillance program to high risk patients for early detection and management of any recurrence.

## Highlights

• AFP/TTV ratio is a feasible surrogate for prediction of HCC recurrence.
• It can help in providing an intensive postoperative surveillance program to high risk patients.
• By knowing the high risk patients for HCC recurrence, we can provide appropriate preventive measures.
• The appropriate preventive measures like loco-regional or new-adjuvant therapy, still need another study to clarify.

## 1 Introduction

Surgical resection for hepatocellular carcinoma (HCC) is considered the most appropriate and curative treatment among the several therapeutic approaches in clinically resectable tumors. However, new lesions can develop as a metachronous HCC recurrence or intrahepatic recurrence of the primary resected tumor [[1], [2], [3]].

Recurrence of HCC is an important factor that may adversely affect patients' survival after liver resection with a devastating outcome. Different factors have been shown to have a significant risk for early or late HCC recurrence including the high preoperative level of alpha fetoprotein (AFP), multiple tumors, large tumor size, macrovascular or microvascular invasion, and type of liver resection. Early identification of these patients with a high risk of tumor recurrence can help in providing an intensive surveillance program that may improve their survival [[4], [5], [6]].

AFP level is commonly used to estimate the severity of tumor burden and as a prognostic measure for the response to different treatments. Some staging systems for HCC showed that using AFP in the scoring system can provide more prognostic benefit in the prediction of the outcome. Total tumor volume (TTV) is another prognostic marker for tumor burden that can accurately predict the outcome by incorporation of the size and number of tumor nodules into one formula [[7], [8], [9], [10]]. The objective of this study was to evaluate the prognostic value of AFP per TTV in the prediction of HCC recurrence after resection.

### 1.1 Patients and methods

We retrospectively identified cirrhotic patients who had a liver resection for HCC in the period between January 2010 and January 2018 at the National Liver Institute, Menoufia University, Egypt. The preoperative, operative and postoperative data were collected and analyzed from the prospective database and the patients' files after the Institutional Review Board (IRB) approval. The study goes with the standards of the Declaration of Helsinki and ethical guidelines and registered in the clinical trial no CTR2000030403. The work was reported in line with the Strengthening the Reporting of Cohort Studies in Surgery (STROCSS) criteria [11].

Patients included in the study had HCC on top of a cirrhotic liver with Child-Pugh score A. Patients with Child B, C score or with any other tumor than HCC were excluded from our study. Patients who had the first 6 months mortality after liver resection were also excluded from the study, for accurate follow up of the recurrence rate. The diagnosis of HCC was done by contrast-enhanced computed tomography (CT) scan or magnetic resonance image (MRI) and confirmed after resection by the histopathological study.

Measurement of the tumor volume was done by the volumetric study in the CT scan or the MRI when available or calculated by using this formula: 4/3 × 3.14 × (maximum tumor radius in cm)³ [9,10,12], TTV of 65.5 cm³ is nearly equal to a tumor with a diameter of 5 cm. Preoperative peak serum AFP level was measured and AFP to TTV ratio was calculated. Patients were divided into 2 groups; a group with AFP/TTV ≤2 and another group with AFP/TTV >2, as the median and mean of AFP/TTV were nearly equal to 2 and further classification of AFP/TTV ≤10 and > 10 was done for early detection of HCC recurrence as reported in different series [13,14].

Follow up of the patients for detection of HCC recurrence was done from the date of surgery up to February 2020 with a median period of follow up 49 months. Follow up was done by AFP and abdominal ultrasound every 3–6 months and contrast-enhanced CT scan or MRI every 6 months in the first year then yearly. HCC recurrence in the first year was considered as early recurrence, and after 1 year was a late recurrence [15]. Risk factors for HCC recurrence and the feasibility of AFP/TTV ratio in prediction of early and late HCC recurrence was investigated. Early postoperative complications were recorded and classified from 0-V according to the Clavien Dindo grades of postoperative complications [16].

The management of recurrent HCC either surgical, interventional or palliative was reported.

### 1.2 Statistical analysis

Statistical analysis was done using SPSS 23 (IBM SPSS Inc., Chicago, IL). Chi-square or Fisher's exact test was used for comparison of the categorical variables. While in continuous variables, the Mann-Whitney *U* test or Kruskal-Wallis test was used for comparison. Recurrence-free survival was calculated by the Kaplan-Meier method, and Log-rank test was applied to compare the difference in survival rates. Logistic regression analysis was appraised in multivariate analysis for risk factors for HCC recurrence. Statistically significant values were considered when the *P* value was less than 0.05.

## 2 Results

A total of 286 HCC patients who received liver resection were included and analyzed in this study. The mean age of overall patients was 59.2 years, and 79.4% of them were male patients. The most common cause of liver cirrhosis was the hepatitis C virus (HCV) (90.2%).

One hundred and two patients had AFP/TTV ≤2, and 184 patients had AFP/TTV >2. Table 1, shows the difference between the 2 groups. In the univariate analysis, there was a significant difference between the 2 groups of patients in the preoperative total bilirubin level (*P* = 0.04), serum AFP level (*P* = 0.001), mean tumor diameter (*P* = 0.01), TTV (*P* = 0.001), operative blood loss (*P* = 0.04), microvascular invasion (*P* = 0.05), and hospital stay (*P* = 0.04).

**Table 1:** Patients demographics, clinicopathological data, operative and post-operative data.

Variable	AFP/TTV ≤2 (n = 184)	AFP/TTV >2 (n = 102)	*P*-value
Age			0.69
Mean ± SD	57.8 ± 4.5	60.2 ± 5.3	
Range	47–73	45–71	
Gender			0.86
Male	148 (80.4%)	79 (77.5%)	
Female	36 (19.6%)	23 (22.5%)	
Etiology of liver disease			0.91
HCV	168 (91.3%)	90 (88.3%)	
HBV	8 (4.3%)	5 (4.9%)	
HCV&HBV	3 (1.6%)	3 (2.9%)	
Other	5 (2.7%)	4 (3.9%)	
Total bilirubin (mg/dl)			0.04
Mean ± SD	0.9 ± 0.48	1.1 ± 0.52	
Range	0.46–1.9	0.55–2.4	
Albumin (g/dl)			0.75
Mean ± SD	3.7 ± 0.4	3.5 ± 0.6	
Range	2.8–4.6	2.8–4.3	
INR			0.32
Mean ± SD	1.1 ± 0.3	1.2 ± 0.31	
Range	0.9–1.6	0.9–1.5	
ALT			0.09
Mean ± SD	51 ± 45	58 ± 76	
Range	29–110	13–137	
MELD			0.22
Mean ± SD	9 ± 3.1	9 ± 4.2	
Range	5–16	6–15	
≤10	125 (67.9%)	74 (72.5%)	0.27
>10	59 (32.1%)	28 (27.5%)	
AFP (ng/ml)			0.001
Mean ± SD	49 ± 196	142 ± 2036	
Range	2.7–634	3–23058	
Tumor number			0.22
1	159 (81.5%)	89 (90.6%)	
2–3	25 (18.5%)	13 (9.4%)	
Tumor site			0.35
Right lobe	116 (63%)	61 (59.8%)	
Left Lobe	53 (28.8%)	30 (29.4%)	
Bilobar	15 (8.2%)	11 (10.8%)	
Tumor diameter (cm)			0.01
Mean ± SD	5.4 ± 3.6	3.5 ± 3.1	
Range	3–12	2.5–7	
TTV (cm3)			0.001
Mean ± SD	74 ± 320	52 ± 45	
Range	15–1200	10–270	
Macrovascular invasion			0.82
Absent	176 (95.7%)	98 (96.1%)	
Present	8 (4.3%)	4 (3.9%)	
Type of operation			0.43
Laparoscopic	11 (6%)	15 (14.7%)	
Open	173 (94%)	87 (85.3%)	
Type of resection			0.30
Anatomical	57 (31%)	25 (24.5%)	
Non anatomical	127 (69%)	77 (75.5%)	
Operative blood loss (ml)			0.03
Mean ± SD	355 ± 310	478 ± 290	
Range	50–2500	100–3000	
Intraoperative blood transfusion			0.18
Mean ± SD	2 ± 1	2 ± 3	
Range	0–12	0–8	
Operative time (min)			0.08
Mean ± SD	230 ± 55	220 ± 65	
Range	178–430	160–220	
Tumor differentiation			0.56
well	38 (20.7%)	17 (16.7%)	
moderate or poor	146 (79.3%)	85 (83.3%)	
Microvascular invasion			0.05
Yes	39 (21.2%)	36 (35.3%)	
No	145 (78.8%)	66 (64.7%)	
Hospital stay (days)			0.04
Mean ± SD	8 ± 6	6 ± 9	
Range	3–22	4–31	
Clavien Dindo grades of complications			0.58
0	83 (45.1%)	45 (44.2%)	
I	43 (23.4%)	27 (26.5%)	
II	30 (16.3%)	19 (18.6%)	
III	22 (12%)	8 (7.8%)	
IV	6 (3.2%)	3 (2.9%)	

SD (slndard deviation), AFP (alpha fetoprotein), TTV (total tumor volume), ALT (alanine aminotransferase), INR (international normalized ratio), MELD (model of end stage liver disease).

**Fig.1:**
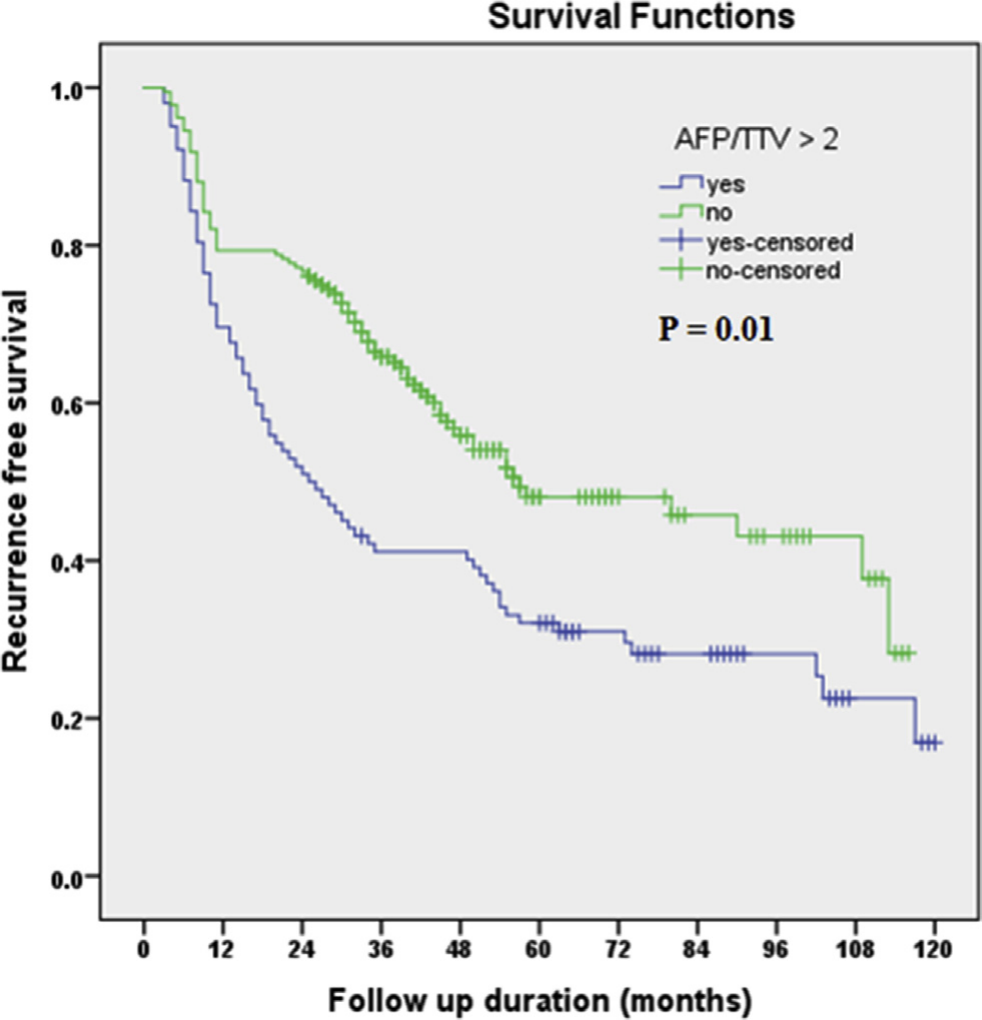
Kaplan-Meier curve for recurrence free survival in patients with AFP/TTV ≤2 and AFP/TTV >2.

The 1, 3, and 5year overall recurrence-free survival rates were 75.9%, 57%, and 42.4% respectively. The 1, 3, and 5year recurrence-free survival in patients with AFP/TTV ≤2 was 79.3%, 65.8%, and 48.1% respectively. While, it was 69.6%, 41.1%, and 32.1% in patients with AFP/TTV >2, Log-rank: (*P* = 0.01) with a significant difference in recurrence-free survival between both groups (Fig. 1).

During the follow up the 1-, 3-, and 5-year tumor recurrence rates were 69 patients (24.1%), 121 patients (43%), and 150 patients (57.6%) respectively.

Table 2 shows the risk factors for early and late HCC recurrence. In univariate analysis the risk factors for 3y HCC recurrence were preoperative total serum bilirubin >1.5 mg/dl (*P* = 0.04), preoperative model of end stage liver disease (MELD) score > 10 (*P* = 0.03), AFP > 400 ng/ml (*P* = 0.04), TTV > 65.5 cm^3^ (*P* = 0.03), AFP/TTV >2 (*P* = 0.01), macrovascular invasion (*P* = 0.001), pathological tumor grades III&IV (*P* = 0.02), and microvascular invasion (*P* = 0.001). AFP/TTV >10 was risk factor for early and late HCC recurrence (*P* = 0.001).

**Table 2:** Early and late HCC recurrence with risk factors for 3 years HCC recurrence.

Variable	All patients	1y Recurrence (n = 69)	3y recurrence	*P*
	(n = 286)		(n = 121)	
Age (years)				0.37
≥60	124	38 (30.6%)	56 (45.2%)	
<60	162	31 (19.1%)	65 (40.1%)	
Gender				0.09
Male	227	50 (22%)	91 (40.1%)	
Female	59	19 (32.2%)	30 (50.8%)	
Total bilirubin (mg/dl)				0.04
≤1.5	204	41 (20.1%)	77 (37.7%)	
>1.5	82	28 (34.1%)	44 (53.7%0	
MELD score				0.03
≤10	199	39 (19.7%)	74 (37.2%)	
>10	87	30 (34.4%)	47 (54%0	
AFP (ng/ml)				0.44
≤100	172	38 (22.1%)	70 (40.7%)	
>100	114	31 (27.2%)	51 (44.7%)	
AFP (ng/ml)				0.04
≤400	217	45 (20.7%)	83 (38.2%)	
>400	69	24 (34.8%)	38 (55.1%)	
Tumor number				0.09
single	248	57 (23%)	101 (40.7%)	
multiple	38	12 (31.6%)	20 (52.6%)	
Tumor size (cm)				0.08
≤5	177	35 (19.8%)	66 (37.3%)	
>5 cm	109	34 (31.2%)	55 (50.5%)	
TTV (cm^3^)				0.03
≤65.5	163	30 (18.4%)	56 (34.4%)	
>65.5	123	39 (31.7%)	65 (52.8%)	
AFP/TTV				0.01
≤2	184	38 (20.7%)	60 (32.6%)	
>2	102	31 (30.4%)	61 (59.8%)	
AFP/TTV				0.001
≤10	245	50 (20.4%)	93 (38%)	
>10	41	19 (46.3%)	28 (68.3%)	
Macrovascular invasion				0.001
Absent	274	62 (22.6%)	112 (40.9%)	
Present	12	7 (58.3%)	9 (75%)	
Type of operation				0.27
Laparoscopic	26	5 (19.2%)	9 (34.6%)	
Open	260	64 (24.6%)	112 (43.1%)	
Type of resection				0.06
Anatomical	82	18 (22%)	27 (32.9%)	
Non anatomical	204	51 (25%)	94 (46.1%)	
Extent of hepatectomy				0.62
Wedge or 1 segment	165	41 (24.8%)	75 (45.5%)	
≥2 segments	121	28 (23.1%)	46 (38%)	
Operative time (min)				0.24
≤300	194	43 (22.2%)	77 (39.7%)	
>300	92	26 (28.3%)	44 (47.8%)	
Operative blood loss				0.15
≤1000	173	39 (22.5%)	66 (38.2%)	
>1000	113	30 (26.5%)	55 (48.7%)	
Blood transfusion			0.12	
Yes	199	52 (26.1%)	90 (45.2%)	
No	87	17 (19.5%)	31 (35.6%)	
Resection margin (cm)				0.09
≤1	110	29 (26.4%)	54 (49.1%)	
>1	176	40 (22.7%)	67 (38.1%)	
Tumor Grading				0.02
I,II	119	22 (18.5%)	35 (29.4%)	
III,IV	167	47 (28.1%)	86 (51.5%)	
Microvascular invasion				0.001
Yes	75	31 (41.3%)	48 (64%)	
No	211	38 (18%)	73 (34.6%)	

SD (standard deviation), HCC (hepatocellular carcinoma), AFP (alpha fetoprotein), MELD (model of end stage liver disease), TTV (total tumor volume).

In the multivariate analysis, independent risk factors for tumor recurrence (Table 3) were AFP/TTV > 2 (HR = 1.62, 95% CI = 1.29–1.98, *P* = 0.042), Macrovascular invasion (HR = 2.03, 95% CI = 2.17–2.38, *P* = 0.021, and microvascular invasion (HR = 1.36, 95% CI = 1.08–1.77, *P* = 0.019).

**Table 3:** Multivariate analysis for independent risk factors for HCC recurrence.

Variable	HR	95% CI	*P*
Total bilirubin >1.5 mg/dl	2.72	1.08–1.91	0.266
MELD >10	2.39	2.17–1.06	0.092
AFP >400 ng/ml	3.18	2.16–1.57	0.281
TTV >65.5 cm^3^	1.94	0.47–2.14	0.065
AFP/TTV >2	1.62	1.29–1.98	0.042
Macrovascular invasion	2.03	2.17–2.38	0.021
Tumor grade III or IV	1.92	2.09–2.71	0.052
Microvascular invasion	1.36	1.08–1.77	0.019

HCC (hepatocellular carcinoma), AFP (alpha fetoprotein), MELD (model of end stage liver disease), TTV (total tumor volume).

For the management of HCC recurrence, 7 patients underwent resection, 38 patients underwent trans-arterial chemoembolization (TACE), 17 patients had radiofrequency ablation (RFA), 2 patients underwent Y90 radioembolization, 8 patients had combined TACE & RFA, 6 patients received chemotherapy (Sorafenib), and the other patients either lost follow-up or died.

## 3 Discussion

It has been suggested that the secretion of AFP correlates directly to cell proliferative activity and tumor size so potentially associated with a more aggressive tumors. Also, it was found that tumor burden and serum level of AFP are well-established markers associated with HCC recurrence [12,13,17].

Some studies in East Asia showed that recurrence of HCC was significant when AFP was >400 ng/ml [13,18], in other study AFP >100 ng/ml was associated with HCC recurrence [19]. A large study on 568 patients reported that the preoperative AFP level was a reliable index for overall survival and disease-free survival post-liver resection [13,20].

Many series provided 1year recurrence rates of 25–32%, 3-year recurrence rates 40–53%, and 5-year recurrence 52–81% [15,21,22], which is a comparable result to our study. Lee et al. reported that 1-, 3-, and 5-year recurrence rates were 29%, 55%, and 68%, respectively. The same study showed that serum international normalized ratio (INR), maximum tumor diameter ≥ 4 cm, number of the tumors, and the presence of macrovascular invasion were important determinants for recurrent HCC [14]. In our study Preoperative bilirubin level, AFP >400 ng/ml, TTV, macro, and microvascular invasion were associated with tumor recurrence in the univariate analysis.

One study evaluated 126 patients with HCC and demonstrated that the independent risk factors for prediction of decreased overall survival and disease-free survival post-resection were large tumor size > 5 cm, high preoperative AFP >400 ng/ml, multiple tumors and vascular invasion [23].

But, there are still controversies in the efficacy of using AFP alone in the prediction of recurrence of HCC post-resection, so other studies identified a more specific means for detection of recurrent HCC like AFP/TTV ratio [10,24,25]. Furihata et al. reported that AFP/TTV >20 was a better prognostic indicator of early HCC recurrence within 6 months after resection than AFP alone [26]. Also, Sharma et al. in their study of 124 patients had the same results [13]. In a large series on 655 patients, Lee and his colleges found that AFP/TTV >1.5 was an independent risk factor for HCC recurrence especially if associated with TTV ≥40 cm^3^, macrovascular invasion, or main tumor diameter ≥ 4 cm [14]. In our study AFP/TTV >2, macrovascular invasion and microvascular invasion were independent risk factors for HCC recurrence in multivariate analysis, also AFP/TTV >10 was a risk factor for early HCC recurrence in the first year after resection.

By knowing these patients with the possibility of a high risk of HCC recurrence, we can try to provide some preventive measures like interventional radiology or new adjuvant chemotherapy instead of surgical resection but this still needs further studies [13,14].

Whether this correlation between AFP and TTV is applicable to be adjusted for patients with previous treatment by loco-regional therapy like chemoembolization or radioembolization rather than resection also has not been studied yet, and need further study and analysis.

The limitations of this study were that it is a retrospective single-center study so liable for statistical bias, the commonest cause of liver cirrhosis and HCC in our study was HCV, which may differ than predominant causes of liver disease and HCC in the other Eastern or Western countries so the tumor biology and secretion of AFP may be affected.

Conclusions: In multivariate analysis, a newly proposed marker of AFP/TTV ratio was an independent risk factor for HCC recurrence than using AFP or TTV alone. It is a feasible surrogate that can be used in selecting the high-risk patients of tumor recurrence that may need intensive postoperative surveillance for early detection and possible treatment or we can consider providing appropriate preventive measures like loco-regional therapy or new-adjuvant chemotherapy for those high-risk patients but further studies are still needed to clarify. This study confirms the previous results of East Asian studies.

## Statement of ethics

The research was conducted ethically in accordance with the World Medical Association Declaration of Helsinki. The patients have given their written informed consent on admission and pre-operative to use their prospective data base and files for research work. The study protocol was approved by the National Liver Institute committee and review board.

## Financial support

No.

## Author contribution

Hazem Zakaria, Anwar Mohamed, Hazem Omar, Nahla Gaballa, actively participated in the preparation, study design, collection of the data and editing of the manuscript. Statistical analysis was done by Hazem Zakaria.

## Provenance and peer review

Not commissioned, externally peer reviewed.

## Declaration of competing interest

No conflicts of interest.
